# Prey Detection and Prey Capture in Copepod Nauplii

**DOI:** 10.1371/journal.pone.0047906

**Published:** 2012-10-29

**Authors:** Eleonora Bruno, Christian Marc Andersen Borg, Thomas Kiørboe

**Affiliations:** Centre for Ocean Life, National Institute for Aquatic Resources, Technical University of Denmark, Charlottenlund, Denmark; Institute of Marine Research, Norway

## Abstract

Copepod nauplii are either ambush feeders that feed on motile prey or they produce a feeding current that entrains prey cells. It is unclear how ambush and feeding-current feeding nauplii perceive and capture prey. Attack jumps in ambush feeding nauplii should not be feasible at low Reynolds numbers due to the thick viscous boundary layer surrounding the attacking nauplius. We use high-speed video to describe the detection and capture of phytoplankton prey by the nauplii of two ambush feeding species (*Acartia tonsa* and *Oithona davisae*) and by the nauplii of one feeding-current feeding species (*Temora longicornis*). We demonstrate that the ambush feeders both detect motile prey remotely. Prey detection elicits an attack jump, but the jump is not directly towards the prey, such as has been described for adult copepods. Rather, the nauplius jumps past the prey and sets up an intermittent feeding current that pulls in the prey from behind towards the mouth. The feeding-current feeding nauplius detects prey arriving in the feeding current but only when the prey is intercepted by the setae on the feeding appendages. This elicits an altered motion pattern of the feeding appendages that draws in the prey.

## Introduction

Copepod nauplii are ubiquitous, abundant and productive in marine waters [1], [2], and they are eaten by many fish larvae [3], [4]. In spite of their central ecological role, there are only a few studies that have examined their feeding ecology and the mechanisms by which they detect and capture prey.

In an early study Storch [5] observed two different modes of feeding: nauplii of the calanoid *Diaptomus gracilis* create a feeding current using the antennae and the mandibles, while nauplii of the cyclopoid *Cyclops strenuus* occasionally grasp food particles. Later, Gauld [6] studied the swimming and feeding of naupliar stages of several calanoid and cyclopoid copepods and found two swimming patterns, a ‘smooth swimming’ due to the motion of the antennae and mandibles, and ‘leaps’ created by all three pairs of appendages. These two motility modes have since been confirmed in several studies, with calanoid nauplii exhibiting both and cyclopoid nauplii only moving in the jerky mode [7]–[11]. The two motility modes correlate with the two feeding modes originally reported by Storch [5]: smoothly swimming nauplii also produce a feeding current that entrain prey, while nauplii that move in the jerky, jump-sink mode are ambush feeders that are non-motile for most of the time while they wait for motile prey to pass by [7]–[10].

Paffenhöfer and Lewis [7] used video recordings for a more detailed description of how nauplii detect and capture prey. In the nauplii of two *Eucalanus* species the phytoplankton prey cells arriving in the feeding current are perceived and elicit a response only when the prey is in the immediate vicinity of the setae of the antennules, presumably using chemical cues. Based on the preference for motile food in *Oithona davisae* [11] Paffenhöfer and Lewis [7] also suggested that ambush feeding nauplii use hydromechanical cues to detect prey. Henriksen et al. [10] described prey attacks in ambush feeding nauplii of *O. davisae*.

According to the descriptions above, copepod nauplii are thus either ambush feeders or they produce a feeding current. It is unclear, however, whether and how feeding-current feeding nauplii can perceive prey remotely. There is a lower size-limit for chemical detection since chemical signals leaking from small cells dissipate almost instantaneously due to molecular diffusion. Empirical evidence suggests a size threshold of about 10 µm [12], but nauplii may feed on much smaller prey [13]. Prey cells arriving in the feeding current may also generate a hydromechanical signal, but the size-threshold for hydromechanical detection is of the order 50 µm [14]. Ambush feeding nauplii may detect motile prey hydrodynamically in much the same way as has been demonstrated for copepodites [15], but it is unclear how the prey can be captured. Attack jumps in calanoid and cyclopoid nauplii were described by [10] using video recordings at 25 Hz. However, such attack jumps should be theoretically ineffective due to the thick viscous boundary layer surrounding the attacking nauplius at these low Reynolds numbers: prey cells will simply be pushed away as the nauplius lunges forward [16].

By means of high-speed video recordings, we here describe the detection and capture of prey in nauplii of two species that are ambush feeders (*Acartia tonsa* (Calanoida), *Oithona davisae* (Cyclopoida)) and one species that produces a feeding current (*Temora longicornis* (Calanoida)). We show that both ambush feeders detect prey remotely and that the prey is pulled towards the mouth by motions of the feeding appendages rather than approached by directs attack jumps. The feeding-current feeding nauplius only detects prey as it touches the setae on the feeding appendages.

## Materials and Methods

### Experimental organisms

Nauplii of *Acartia tonsa*, *Oithona davisae* and *Temora longicornis* were collected from our continuous laboratory cultures that were maintained at 30 Practical Salinity Units (PSU) and 16°C (*A. tonsa*, *T. longicornis*) or 22°C (*O. davisae*). We used 3 different species of motile prey (Table 1): *Rhodomonas salina* (cryptophyte, Equivalent Spherical Diameter, ESD 7 µm), *Oxyrrhis marina* (heterotrophic dinoflagellate, ESD 17 µm) and *Heterocapsa triquetra* (dinoflagellate, ESD 14 µm).

**Table 1:** Characteristics of the observed nauplii and of the attacks.

Species	Naupliusstage	Lengthmm	Prey species	Distance of prey from the tip of nearest antennulemm	Distance of prey from setae on nearest antennulemm	Attack/Capture durationms	Maximum attack velocitymm s^−1^	Average attack velocitymm s^−1^	Handling timems	Boundary layer thicknessmm
*A. tonsa*	NII-NIII	0.209±0.004	*R. salina*	0.111±0.03	0.117±0.07	42±14	52±23	17.8±5	343.5±28	0.13
	NIV-NVI	0.234±0.02	*R. salina*	0.197±0.1	0.179±0.12	44±25	57.8±16	18.9±8	206.7±121	0.12±0.05
*O. davisae*	NII-NIII	0.106±0.002	*O. marina*	0.09±0.05	**-**	29±15	38±9	11.6±6	360.7±74	0.097±0.04
	NIV-NVI	0.125±0.011	*O. marina*	0.067±0.04	**-**	14.6±11	30.6±7	12.3±4	232.3±81	0.162±0.1
*T. longicornis*	NIII	0.112±0.004	*R. salina*	**-**	**-**	92.5±40	-	-	-	-
	NIV-NVI	0.210±0.07	*R. salina*	**-**	**-**	104.7±38	-	-	226±171	-
	NIV	0.166±0.04	*H .triquetra*	**-**	**-**	233.5±88	-	-	626	-

Species of the experimental nauplii, developmental stage and size (±SD) of the observed nauplii of *Acartia tonsa*, *Oithona davisae* and *Temora longicornis*, prey species used, distance of prey from the tip of nearest antennule and from setae on nearest antennule, attack (capture for *T. longicornis*), maximum and average attack velocity, handling time, thickness of the viscous boundary layer.

### Observations

We observed the feeding behaviour of nauplii in either a 60 ml polycarbonate aquarium or a 4 ml glass cuvette (1 cm×1 cm×4 cm) containing 0.2 µm filtered seawater (30 PSU) and prey organism at a super-saturating concentration. We had 1–10 nauplii ml^−1^. All experiments were conducted in a temperature-controlled room at 16°C for *A. tonsa* and 22°C for *O. davisae* and nauplii were allowed to acclimate for ca. 1 hour before filming. *A. tonsa* nauplii were offered *R. salina*, *O. davisae* nauplii were offered *O. marina*, and *T. longicornis* nauplii were offered both *H. triquetra* and *R. salina* (Table 1).

Feeding events were recorded using a high-speed, high resolution (1024×800 pixels) Phantom v210 digital video-camera, at a frame rate of 2000 frames s^−1^ for *A. tonsa* and *O. davisae*, and 2200 frames s^−1^ for *T. longicornis*. The camera was equipped with lenses to yield a field of view of 7.8 or 31.4 mm^2^. Illumination was provided by a halogen bulb that back-lit the aquarium. We recorded prey capture events that occurred by chance within the focal plane. Altogether we recorded 9 events for *A. tonsa*, 9 events for *O. davisae* and 10 events for *T. longicornis*.

**Figure 1:**
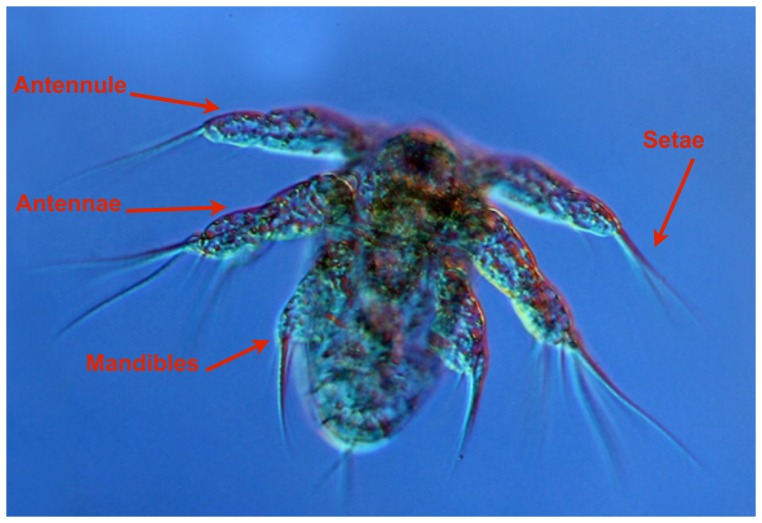
Appendages analyzed. *(Acartia tonsa* nauplius showed as example): antennules (A1), antennae (A2) and mandibles (Md).

**Figure 2:**
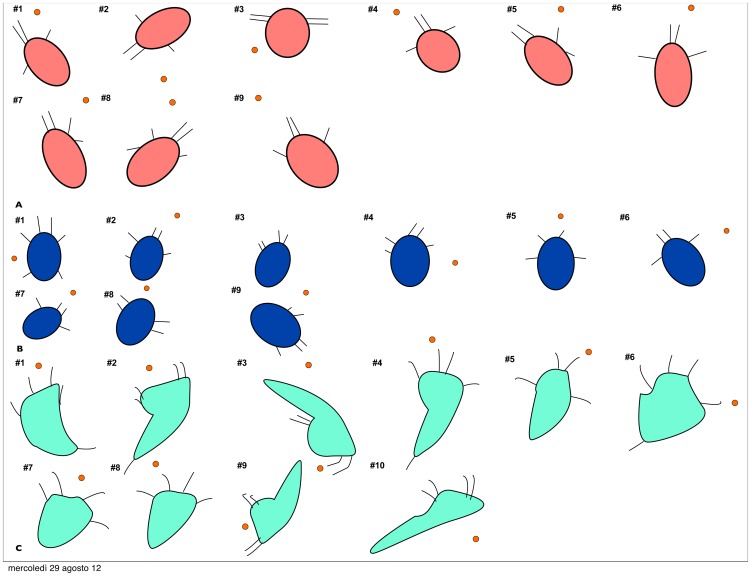
Position and orientation of the nauplii. Schematic drawing of the position and orientation of the nauplii and their prey (represented by small red dots) immediately prior to attack: (A) *Acartia tonsa* and *Rhodomonas salina*, (B) *Oithona davisae* and *Oxyrrhis marina*, (C) *Temora longicornis* and *R. salina* (*Heterocapsa triquetra* in #3 and #7), not in scale.

The feeding events were analysed frame-by-frame using the shareware ImageJ. We digitized the position of the nauplius (tip of head, end of body), the position of the tips of the appendages (3 pairs in *A. tonsa*, up to 4 pairs in *O. davisae* and *T. longicornis*) (Fig. 1), and the position of the prey (Fig. 2). The positions were subsequently transformed into a coordinate system that had its origin at the tip of the head and the z-axis aligned with the length of the body. All distances are measured in 2-dimensional projections and are therefore conservative.

In addition, the following parameters were estimated: (1) *Prey detection distance*, defined as the distance to the prey at the time when the nauplius first reacts (we recorded both the distance from the tip of the nearest antennules and the distance from the setae on the nearest antennules), (2) *attack distance* defined as the distance covered by the nauplius from the detection to the capture of the prey, (3) *attack duration* defined as the time from detection to capture of the prey, (4) *prey handling time* defined as the time from capture of the prey until the nauplius has resumed normal swimming, or has stopped moving, and (5) maximum and average body velocity during the attack.

Depending on the position of the nauplius and its prey relative to the camera, not all parameters could be measured for all prey capture events. Also, not all the measurements listed above applied to the nauplii of *T. longicornis* as they appeared unable to detect prey remotely.

## Results

### Detection, capture, and handling of prey

#### Acartia tonsa

**Figure 3:**
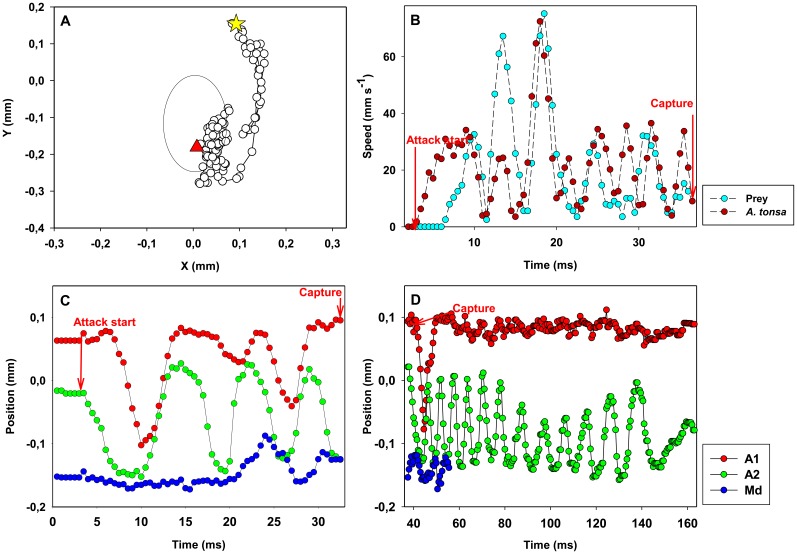
*Acartia tonsa*: example of prey captures and X-position of the appendages. *Acartia tonsa* and its prey (*Rhodomonas salina*). (A) Position of prey (circles) relative to the nauplius (the big oval) from detection of the prey (star) till the prey has disappeared from view and is captured (triangle). Time interval between dots: 0.5 ms. The change in relative position of the prey is due to the combined effect of the nauplius moving forward and the prey being pushed away; the prey does not swim significantly during the attack. (B) Velocity of predator and prey as a function of time. C,D.Temporal variation in X-position of appendages during prey capture (C) and prey handling (D).

The majority of the analysed captures were elicited by remote detection of the prey, i.e., with no physical contact between the nauplius and the prey. The average detection distance was a little less than one body length (Table 1). In two records (capture #3 and #5) it was unclear whether the nauplius actually touched the prey prior to attack. At detection, the prey was positioned anterior or lateral to the nauplius (Fig. 2A, Fig. 3A).

**Figure 4:**
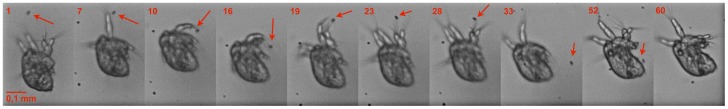
Prey capture in *Acartia tonsa*. Time series of still images with frame numbers indicated (consecutive frames are 0.5 ms apart). Arrows point towards the prey. In the last image the prey has been captured.

A typical attack begins with the nauplius jumping towards the prey by sequentially striking two of the appendage pairs (Fig. 3, 4 and movie S1). It starts with the antennae (A2) that also deliver the main propulsion power, followed by the antennules (A1). During the forward jump the mandibles (Md) do not move. After the initial power stroke, the antennules (A1) bend in the recovery stroke, followed by another power stroke of A1 and A2. As the nauplius lunges towards the prey, accelerating to a speed of several hundred body lengths s^−1^ (Fig. 3B), the prey is pushed away and the nauplius typically swims past the prey with several strokes of the appendages while the prey remains at a substantial distance (Fig. 3C, Fig. 4). Finally, with counter movements of A2 and Md, circa 22 ms after the initiation of the attack, the nauplius creates a flow that pulls the prey forwards towards the mouth from a position posterior to the nauplius, and the prey is captured after ca. 33 ms (Fig. 3D). There are some variations to this pattern and the duration of the attack varies more than fivefold (Table 1). The prey is never approached directly, but rather passed as the nauplius jumps forward, and the prey is finally pulled towards the mouth by a temporary current generated by A2 and Md. Capture #9 represents an exception to the typical “prey by-pass – feeding current from behind” mode: the nauplius seems to approach the prey directly, no temporary current is generated, and the prey is immediately ingested.

Following a capture, prey is handled over a 100 to 400 ms period (Table 1). The handling can be divided in two phases (Fig. 3D). During the first phase, the nauplius moves A2 and Md back and forth in counter-phase while the prey is handled. The A1 make only small movements. In the second phase, Md are motionless, while A2 are beating vigorously at about 130 Hz. A1 are still making small movements. The handling is concluded when the appendages are again in their resting mode. In two cases (capture #4 and #9) a grooming-like movement is observed after the end of the handling. It consists of counter-movements of A2 and Md while the copepod is stationary, and it lasted 20 ms in capture #4 and 156 ms in capture #9.

#### Oithona davisae

**Figure 5:**
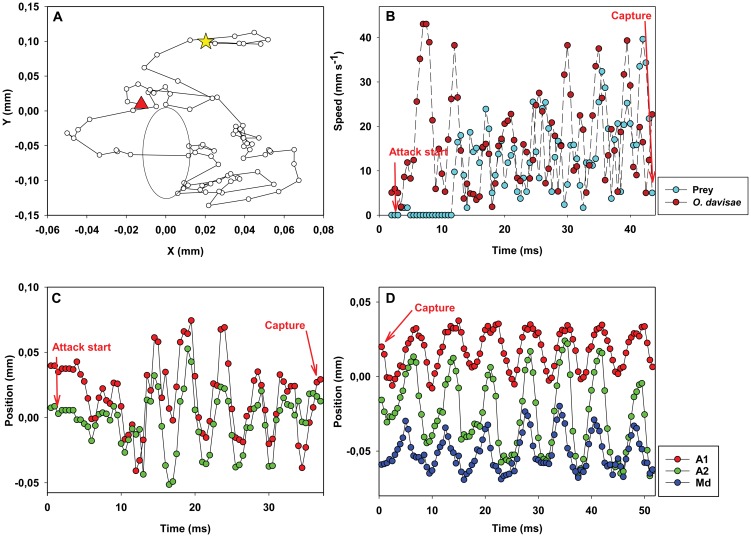
*Oithona davisae*: example of prey captures and X-position of the appendages. *Oithona davisae* and its prey (*O. marina*). (A) Position of prey (circles) relative to the nauplius (the big oval) from detection of the prey (star) till the prey has disappeared from view and is captured (triangle). Time interval between dots: 0.5 ms. The change in relative position of the prey is due to the combined effect of the nauplius moving forward and the prey being pushed away; the prey does not swim significantly during the attack. (B) Velocity of prey and predator as a function of time. C,D.Temporal variation in X-position of appendages during prey capture (capture #3) (C) and prey handling (capture #7) (D).

In at least 6 out of 9 attacks, remote detection was involved in the capture of the prey, as there was no physical contact between the nauplius and the prey prior to the nauplius reacting. The average reaction distance was a little more than half a body length (Table 1). In two records (capture #5 and #8), it was not possible to see both the A1 due to the position of the nauplius, while in attack #9 the nauplius sinks over the prey, thus remote detection is not discernible. All the attacks were commenced when the nauplius was motionless. At detection, the prey was located anterior, lateral, or even posterior to the nauplius (Fig. 2B, Fig. 5A).

**Figure 6:**
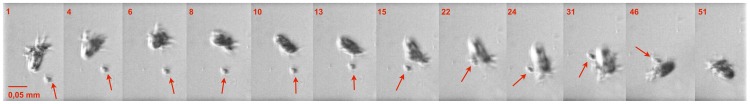
Prey capture in *Oithona davisae*. Time series of still images with frame numbers indicated (consecutive frames are 0.5 ms apart). Arrows point towards the prey. In the last image the prey has been captured.

The attack begins when the nauplius jumps by sequentially striking the three anterior pairs of appendages at about 160 Hz. In the later developmental stages the first maxillae (Mxl) are present but not moving (Fig. 5, 6 and movie S2). Generally the antennae (A2) start the beat cycle, immediately followed by the antennules (A1), then the mandibles (Md). The mandibles begin the recovery phase while A2 and A1 are still in the beat phase. In half of the attacks the nauplii first make a lateral turn with an asymmetrical power stroke. After the initial turn, the nauplius jumps past the prey at a speed ranging from 20 to more than 100 body lengths s^−1^ (Fig. 5B), making a turn around the prey. The attack time varies substantially but averages 20 ms (Table 1). In some recordings the nauplius demonstrated great manoeuvrability, turning while jumping, and orienting itself relative to the prey: this happened when the prey was detected in a position posterior to the nauplius (Fig. 6). Despite the apparently awkward manoeuvres, the prey was usually captured in the first attempt.

Prey handling started immediately after prey capture and lasted on average 275 ms (Table 1), substantially longer than in *A. tonsa*. Prey handling was also different from that in *A. tonsa*.

*O. davisae* beats all appendages sequentially and repeatedly at ca. 140 Hz (e.g. capture #7 Fig. 5D). A2 begins the beat cycle, followed by A1, and then Md. The nauplius hover in the water while handling the prey, or moves very slowly forward, which makes prey handling distinctly different from relocation jumps.

#### Temora longicornis

**Figure 7:**
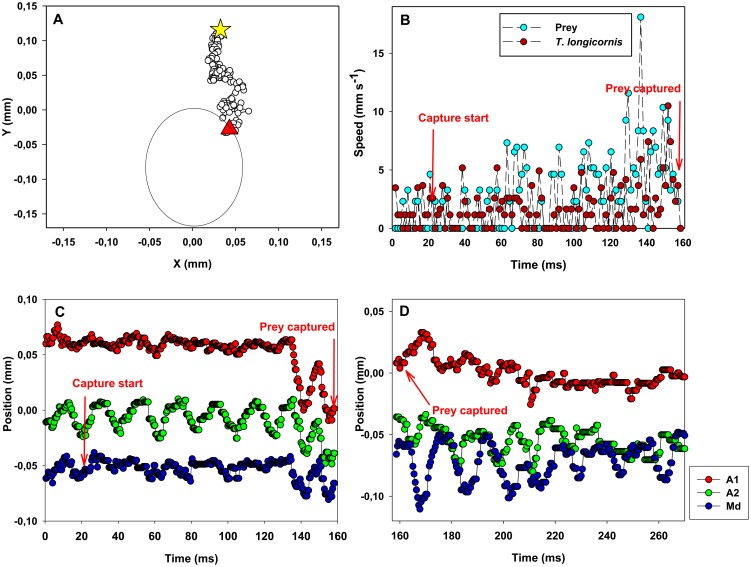
*Temora longicornis*: example of prey captures and X-position of the appendages. *Temora longicornis* and its prey (*R. salina*). (A) Position of prey (circles) relative to the nauplius (the big oval) from detection of the prey (star) till the prey has disappeared from view and is captured (triangle). Time interval between dots: 0.45 ms. The change in relative position of the prey is due to the combined effect of the nauplius moving forward and the prey being pushed away; the prey does not swim significantly during the attack. (B) Velocity of prey and predator as a function of time. C, D. Temporal variation in X-position of appendages during prey capture (C) and prey handling (D).

The observed captures were apparently not elicited by remote detection. The nauplius only responds to the presence of the algae once the prey touches the setae of one of the appendages. Consequently, it is not possible to indicate a detection distance (Table 1). At encounter, the prey was always anterior to the nauplius (Fig. 2C, 7A).

**Figure 8:**
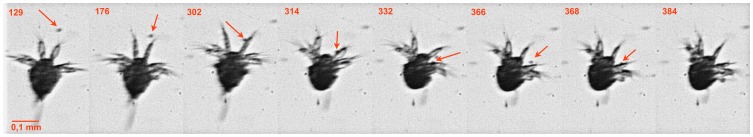
Prey capture in *Temora longicornis*. Time series of still images with frame numbers indicated (consecutive frames are 0.45 ms apart). Arrows point towards the prey. In the last image the prey has been captured.

The nauplii generate a feeding current by continuously vibrating the appendages at 30 Hz (Fig. 7, 8). The feeding current also pulls the nauplii slowly through the water. The beat cycle is initiated by the mandibles (Md), followed by the antennae (A2) and then the antennules (A1). Also, the antennules rotate back and forth around their own axis, with left and right A1 counter-rotating. Md and A1start the recovery phase approximately at the same time and simultaneous with the power stroke of A2 that thus moves in counter phase. Md complete the recovery phase slightly before A1 (Fig. 7C).

Prey are entrained in the feeding current that accelerates in towards the nauplius to an average peak velocity of 3.1 mm s^−1^ (Fig. 7B). The prey is generally touched with the setae on one of the A1. Prey detection elicited different types of responses. In 4 events there was no apparent reaction to the perception of the prey, which is just pulled in and disappears (eaten). In one event the nauplius reacted immediately to the prey, backed somewhat for circa 15 ms, then resumed its previous position, and finally pulled in the prey with the feeding current. In five events (Fig. 8) there was a time lag of on average 60 ms, before the nauplius backed without any further repositioning during the subsequent capture. In all captures, A2 and Md create a flow that carries the prey towards the nauplius mouth. The initial stroke during the creation of this capture flow is given by Md, followed by A2 after circa 2 ms (see movie S3). The time interval between the reaction to the prey and the capture is 58 ms on average. During the entire process the antennules rotate around their length axes but do not beat; a motion that presumably keeps the nauplius in a fixed position. The prey was usually captured in the first attempt.

The handling time was on average 306 ms (Table 1). It is not always possible to define precisely when the prey handling is completed because handling is not distinctly different from generating a feeding current. Therefore, we define handling as the time from when the prey disappears to the start of a sinking period or a jump. In some movies it was not clear whether the handling period was concluded or not, because the nauplius continued to swim creating a feeding current. The mandible initiates the handling by a recovery stroke, then A1 start a beat cycle, followed by A2 (Fig. 7D). The pair of A1 moves only a little, limiting the forward motion of the nauplius.

## Discussion

### Detection

The ambush feeding nauplii of *Acartia tonsa* and *Oithona davisae* both detect their prey remotely. This is in accordance with earlier observations on ambush feeding copepodites and adults [15], [17]–[19]. The detection distances recorded in this study, 0.1–0.2 mm from the antennules, are consistent with the cue for prey detection being hydromechanical. The fluid disturbance generated by a self-propelled prey can be described by a stresslet [14] and the distance at which it can be perceived by a rheotactic predator (*R*) can, to first order, be estimated as *R*≈*a*√6πν/u* where *a* is the radius of the prey, *v* is its swimming velocity, and u* the threshold fluid velocity required for perception [19]. If we assume u* = 20×10^−3^ mm s^−1^ [20], *v* = 20×10^−2^ mm s^−1^ for a 10^−2^ mm radius flagellate prey, then *R*∼0.14 mm, similar to that observed. This calculation suggests that both of the ambush feeding nauplii sense their prey hydromechanically, as has been suggested for adult, ambush feeding copepods [16], [21].

*Temora longicornis* nauplii appear to detect prey arriving in the feeding current only as the prey makes direct contact with the setae on the antennules or on the antennae. This is in agreement with the observations of [7] on the feeding-current feeding *Eucalanus crassus* and *E. pileatus*, that responded to the prey (*Talassiosira weissflogii* 12 µm and *Rhizosolenia alata* 25–40 µm by 150–500 µm) when they were at a distance 0.1 or 0.3 mm, respectively, away from the tips of their antennae and mandibles. Paffenhöfer and Lewis [7] assumed that the nauplii detected the prey with the use of chemoreceptors on the distal parts of setae. The setae measure 0.1 to 0.3 mm, which means that these nauplii sense the prey in the immediate proximity of the setae [7]. It is not obvious from their observations whether chemical or tactile sensing is involved in detection. Nauplii of *T. longicornis*, however, do not rely on chemo-detection. *Temora longicornis* nauplii cannot register the presence of the prey until they touch it with the setae. This is in accordance with the lower-size limit (about 10 µm) for the individual detection of the prey by the use of chemical cues [12].

### Clearance rate

The ambush feeding nauplii depend on the prey swimming into the perceptive sphere of the nauplius, and one may ask if ambush feeding yields sufficiently high clearance rates for the nauplii to sustain a living. Ambush feeding nauplii only target actively motile prey while feeding-current feeders can scan larger volumes of water for prey [10]. The estimate of the clearance rate depends strongly on the swimming speed and motility pattern of the prey, i.e. whether the prey at the spatial scale of the nauplius swims along a straight line (ballistically), or swims along a convoluted path (diffusively) [22]. If the prey swims along a more or less straight line, then the clearance rate can be estimated as π(*R*+*r*)^2^Δ*v*, where *r* is the radius of the nauplius, *R* the perception distance and Δ*v* the velocity difference between the nauplius and the prey (typically dominated by the sinking speed of the nauplius). The clearance rates estimated this way, taking *R* as the distance from the tip of the nearest antennule to the prey, and *v* as the sinking speed of the nauplius, are 2.5 ml d^−1^ for *A. tonsa* nauplii and 0.3 ml d^−1^ for *O. davisae* nauplii, respectively. The values of *v* for *A. tonsa* are taken from [9], and from [10] for *O. davisae*).

The specific clearance rate required to live in the ocean is on the order 10^6^ body volumes d^−1^ [23].The specific clearance rates for the two species are 1.5×10^6^ d^−1^ (*A. tonsa*) and 1.7×10^6^ d^−1^ (*O. davisae*). Thus, the ambush feeding strategy yields sufficiently high clearance rates for the nauplii to survive. This is also supported by the similar maximum growth rates of jump-sinking and cruising nauplii [24]–[28] which imply food clearance rates of similar magnitudes [11].

We can similarly compute absolute and specific clearance rates for the feeding-current feeding nauplii of *T. longicornis* using the same equation, now interpreting Δ*v* as the peak feeding current velocity, and *r* the radius covered by the antennules. The average absolute clearance rate is 11 ml d^−1^, similar to that measured directly [29], while the specific clearance rate is 1.6×10^6^ d^−1^.

### Capture

Copepod nauplii operate at low Reynolds numbers and are therefore surrounded by a viscous boundary layer of thickness *δ*. During the attack jump, the prey will be pushed away by this boundary layer, and the attacking copepod cannot therefore get closer to the prey than the thickness of its boundary layer. At rest, before the nauplius initiates an attack jump, there is no boundary layer. As the nauplius accelerates towards the prey, the boundary layer will grow diffusively with time (*t*) as ∼ (*νt*)^−1/2^, where *ν* is the viscosity, and reach a thickness ∼ (ν*T*)^−1/2^ at the conclusion of the jump at time *T*. In copepodites, the jump duration is so short that the copepod can reach the prey before the viscous boundary layer grows too thick relative to the size of the copepod, but this is not feasible in copepods smaller than about 0.25 mm [16]. Consequently, attacking nauplii push the prey away, such that the distance to the prey always exceeds the boundary layer thickness (Table 1). Direct attacks are, thus, not possible, but the attack jump serves to position the nauplius relative to the prey such that the prey can subsequently be pulled in by the temporary feeding current generated by back-and-forth movements of A2 and Md in *Acartia tonsa*.

*Oithona davisae* nauplii similarly do not jump directly towards the prey, but the nauplius makes intermediate turns and repositioning jumps before capturing the prey. The resolution of our observations did not allow us to document a temporary feeding current, as in *A. tonsa*, although this may be the way that the prey is eventually captured.

*Temora longicornis* nauplii produce a feeding current and translate only slowly through the water, similar to what has previously been described for the same and other *Temora* species [8], [9]. This near hovering feeding-current strategy is adopted by many adult copepods as well as many other plankters, presumably because it is more efficient than cruise feeding [30], [31]. For the same amount of energy, a hovering copepod can scan a larger volume of water than a cruising one [32].

### Prey escape

Ambush feeding nauplii only target motile prey and these prey may be evasive. Many protist prey can perceive fluid disturbances caused by predators, and respond by rather powerful escape jumps [33]. The fluid signal generated by a slow-sinking ambush feeding nauplius is too low to elicit an escape response in protists [34]. Therefore, predator detection and subsequent escapes by protozoans should be less efficient when the predator is an ambush feeder [33]. However, as the nauplius initiates its attack, it generates a significant fluid disturbance that may signal the attack to the prey but that also pushes the prey around. The average velocity of the prey as it is entrained in the flow field generated by the attacking nauplius (7–12 mm s^−1^, fig. 3B and 5B) exceeds known jump speeds of protists (<5 mm s^−1^) [33], and thus prevents the prey form escaping. Hence, attack success in ambush feeding nauplii may be high, even when the prey is evasive. Escape jumps however, may be an efficient defence mechanism when the predator produces a feeding current, like the nauplii of *T. longicornis* [33]. The prey used for *T. longicornis* nauplii in the present experiments, *R. salina*, however, cannot perform escape jumps, and its swimming speed, 0.15 mm s^−1^ [35] does not allow it to escape the feeding current.

## Supporting Information

Video S1 ***Acartia tonsa*** **attack 7 (slow motion 1∶80).**
(M4V)

Video S2 ***Oithona davisae*** **attack 3, attack 7 (slow motion 1∶80).**
(M4V)

Video S3 ***Temora longicornis*** **capture 5 (slow motion 1∶90).**
(M4V)
